# Protein profiling in systemic sclerosis patients with different pulmonary complications using proteomic antibody microarray

**DOI:** 10.1186/s13075-024-03267-z

**Published:** 2024-01-17

**Authors:** Jing Huang, Honglin Zhu, Sijia Liu, Mengtao Li, Yisha Li, Hui Luo, Xiaoxia Zuo

**Affiliations:** 1 Department of Rheumatology, Xiangya Hospital, Central South University, Provincial Clinical Research Center for Rheumatic and Immunologic Diseases, Changsha, China; 2 National Clinical Research Center for Geriatric Disorders, Xiangya Hospital, Changsha, China; 3 Department of Rheumatology, Peking Union Medical College Hospital, Peking Union Medical College & Chinese Academy of Medical Science, National Clinical Research Center for Dermatologic and Immunologic Diseases, Ministry of Science & Technology, Key Laboratory of Rheumatology and Clinical Immunology, Ministry of Education, State Key Laboratory of Complex Severe and Rare Diseases, No.1 Shuaifuyuan, Beijing, 100730 China

**Keywords:** Systemic sclerosis, Pulmonary arterial hypertension, Interstitial lung disease, Calcitonin, Sclerostin/SOST

## Abstract

**Background:**

Pulmonary arterial hypertension (PAH) and interstitial lung disease (ILD) are leading causes of systemic sclerosis (SSc)-related death. In this study, we aimed to identify biomarkers for detecting SSc pulmonary complications that are mild and in the early stages to improve the prognosis.

**Methods:**

We screened for serum biomarkers using a proteomic antibody microarray that simultaneously assessed 1000 proteins. Differentially expressed proteins were further verified using ELISA. Finally, we performed a correlation analysis using clinical data.

**Results:**

We identified 125 differentially expressed proteins, of which calcitonin, sclerostin (SOST), CD40, and fibronectin were selected for further verification. Serum calcitonin and SOST levels were significantly elevated in all SSc pulmonary complication subgroups, whereas serum calcitonin levels were higher in the SSc with PAH subgroup than in the SSc without PAH and ILD subgroup. Serum SOST levels were possibly associated with the presence of ILD and positively related to the presence of cardiac and gastrointestinal involvement. Serum CD40 and calcitonin levels appeared to be positively related to the presence of renal involvement, and serum calcitonin was also positively related to the presence of gastrointestinal involvement.

**Conclusions:**

This study indicated that serum calcitonin and SOST levels may be promising biomarkers for SSc-related PAH and ILD, respectively. Further research is needed to verify this result and understand the underlying mechanisms.

**Supplementary Information:**

The online version contains supplementary material available at 10.1186/s13075-024-03267-z.

## Background

Systemic sclerosis (SSc) is a multi-organ involved autoimmune disease with one of the highest mortality rates among connective tissue diseases [1]. Pulmonary arterial hypertension (PAH) and interstitial lung disease (ILD) remain the leading causes of SSc-related deaths, representing 30–40% of mortalities [2–5]. High mortality rates are largely due to the lack of specific treatments for end-stage tissue fibrosis [1]. Therefore, early diagnosis and treatment may improve outcomes in patients with SSc, especially those with pulmonary complications (including PAH and ILD). We aimed to identify early biomarkers of pulmonary complications in patients with SSc to detect pulmonary complications in its mild form or at an early stage.

Proteomic antibody microarrays allow the simultaneous analysis of many proteins in patient serum samples. In this study, we performed a proteomic chip-based analysis to identify differentially expressed serum proteins among 1000 proteins in patients with SSc and PAH or ILD. Finally, we identified 125 differentially expressed proteins (DEPs), of which calcitonin and sclerostin (SOST) levels were confirmed to be differentially expressed by further verification. These proteins may be promising early biomarkers for SSc and pulmonary complications in patients with SSc.

## Methods

### Study population and samples

This study was approved by the Medical Ethics Committee of Peking Union Medical College Hospital (PUMCH) and the Ethics Committee of the European League Against Rheumatism Scleroderma Trial and Research Group (EUSTAR). Patients with SSc included in this study received a first and confirmed diagnosis at PUMCH between 2009 and 2016 and were prospectively registered in the EUSTAR database. The patients fulfilled the 1980 American Rheumatism Association classification criteria for SSc. All participants (patients with SSc and healthy controls [HCs]) provided informed consent, and their serum samples at baseline were collected and stored at − 80 °C.

### Proteomic antibody microarray

The antibody array (RayBio® L-Series human antibody array 1000 kit; RayBiotech, Norcross, GA, USA) assessed the serum expression levels of 1000 proteins, following the manufacturer’s instructions. The GenePix 4000B microarray scanner (Molecular Devices, LLC; 1311 Orleans Drive Sunnyvale, CA, USA) captured and quantified the signals. We calculated spot intensities by subtracting the background and normalizing the data to the positive controls on individual slides. Independent-sample *t*-test and fold change values were used to determine the DEPs between each SSc subgroup and the HC group. DEPs were confirmed by a *p*-value below 0.05, and a fold change over 1.2 (upregulated) or under 0.8 (downregulated).

### Bioinformatics analysis

We used the R package clusterProfiler to analyze the DEPs in each SSc subgroup. In the Gene Ontology (GO) functional analysis, we displayed the top 10 enriched biological activities in which these proteins primarily participated for each SSc subgroup.

### Evaluation of the serum levels of calcitonin and SOST in additional individuals

Serum calcitonin and SOST levels were measured using Human Calcitonin ELIST Kit (LifeSpan BioSciences Inc.) and Human SOST Immunoassay (R&D Systems), respectively, following the manufacturer’s instructions. The optical density of each well was determined at 450 nm using a microplate reader. The concentration was calculated from the optical density according to the manufacturer’s instructions.

### Statistical analysis

All data were processed using the Statistical Package for the Social Sciences version 21.0 (SPSS, Chicago, IL, USA). An independent-sample *t*-test was used to analyze normally distributed continuous variables. Correlation analysis between serum protein levels and clinical data was performed using Pearson’s correlation analysis (for normally distributed continuous variables of clinical data) and the point biserial correlation test (for classified variables of clinical data). In the correlation analysis, the degree of correlation was higher when the correlation coefficient was close to 1.0 or − 1.0. For all statistical analyses, a two-tailed *p*-value < 0.05 was considered statistically significant.

## Results

### Serum protein profiles in patients with SSc with different pulmonary complications

#### Characteristics of study participants

In total, 15 patients with SSc who were not receiving treatment were included in the protein microarray study: in the subgroup of SSc with PAH (*n* = 5), PAH was defined as a mean pulmonary arterial pressure (mPAP) no less than 25 mmHg and pulmonary arterial wedge pressure (PAWP) no more than 15 mmHg in the right heart catheter, and ILD was excluded by high-resolution computerized tomography (HRCT); in the subgroup of SSc with ILD (*n* = 5), ILD was diagnosed using HRCT, and PAH was excluded when the tricuspid regurgitation velocity (TRV) was no more than 2.8 m/s on echocardiography; in the subgroup of SSc without PAH and ILD (*n* = 5), PAH and ILD were respectively excluded by echocardiography and HRCT. Five age- and sex-matched HC volunteers were recruited and their blood samples were donated. Twenty participants were recruited for the antibody microarray study. Detailed information on these participants is provided in Table 1.

**Table 1:** Demographic information of study participants for high-throughput antibody microarray screening

Group	No	Age (year)	Gender	Course (year)	Subtype	Modified Rodnan Skin Score	Right heart catheter		Echocardiography	ILD in HRCT
							**mPAP (mmHg)**	**PCWP (mmHg)**	**TRV (m/s)**	
**SSc-PAH**	1	56	Female	1.38	dcSSc	15	65	8	4.7	No
	2	53	Male	3.78	lcSSc	4	59	11	4.7	No
	3	50	Female	17.36	lcSSc	2	59	15	4.1	No
	4	43	Female	7.07	lcSSc	2	66	10	5.0	No
	5	34	Female	1.00	lcSSc	4	43	10	-	No
**SSc-ILD**	6	39	Male	12.70	dcSSc	17	-	-	2.2	Yes
	7	36	Female	4.40	lcSSc	2	-	-	2.3	Yes
	8	22	Female	4.69	lcSSc	9	-	-	2.3	Yes
	9	55	Female	20.10	lcSSc	7	-	-	2.5	Yes
	10	58	Female	12.80	lcSSc	7	-	-	2.8	Yes
**SSc-nonPAH-nonILD**	11	34	Female	0.86	dcSSc	15	-	-	2.3	No
	12	35	Female	2.66	lcSSc	2	-	-	2.5	No
	13	47	Male	1.45	dcSSc	17	-	-	2.8	No
	14	41	Female	13.38	lcSSc	8	-	-	2.2	No
	15	52	Female	10.12	lcSSc	5	-	-	2.5	No
**HC**	16	41	Male	-	-	-	-	-	-	-
	17	26	Female	-	-	-	-	-	-	-
	18	53	Female	-	-	-	-	-	-	-
	19	43	Female	-	-	-	-	-	-	-
	20	52	Female	-	-	-	-	-	-	-

*PAH* Pulmonary arterial hypertension, *ILD* Interstitial lung disease, *HC* healthy control, *dcSSc* diffuse cutaneous systemic sclerosis, *lcSSc* limited cutaneous systemic sclerosis, *mPAP* mean pulmonary arterial pressure, *PCWP* pulmonary capillary wedge pressure, *TRV* tricuspid regurgitation velocity, *HRCT* high resolution computed tomography

#### Differentially expressed proteins in each SSc subgroup

**Fig. 1:**
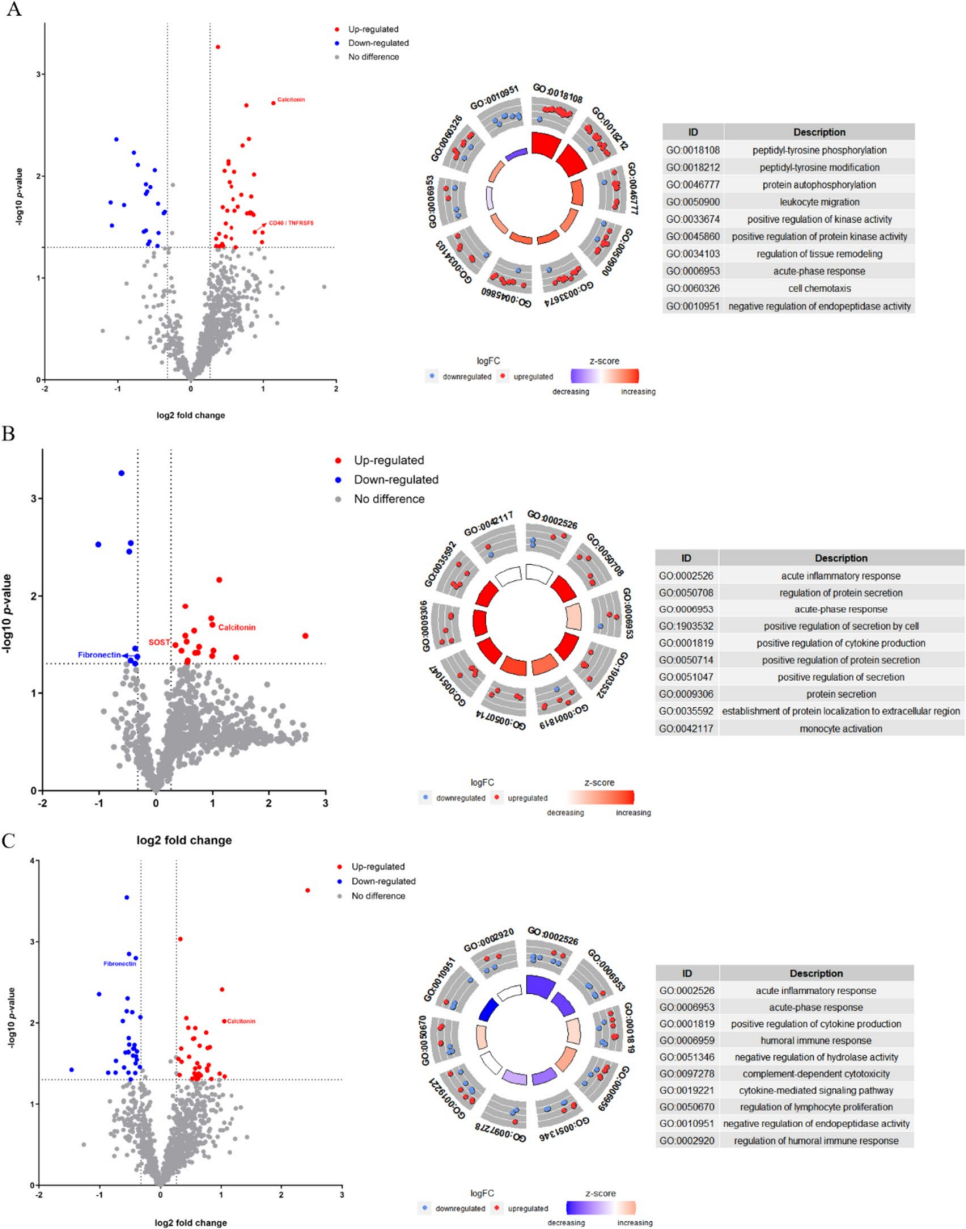
Differentially expressed proteins and functional analysis in each SSc subgroup. Comparison of each SSc subgroup (**A**–**C** for SSc with PAH group, SSc with ILD group, and SSc without PAH and ILD group successively) with HCs. Volcano plots on the left show differentially expressed proteins with a *p*-value < 0.05, fold change > 1.2 or < 0.8. The circle diagrams on the right show the top 10 terms based on a *p*-value < 0.05 in the GO functional analysis

**Fig. 2:**
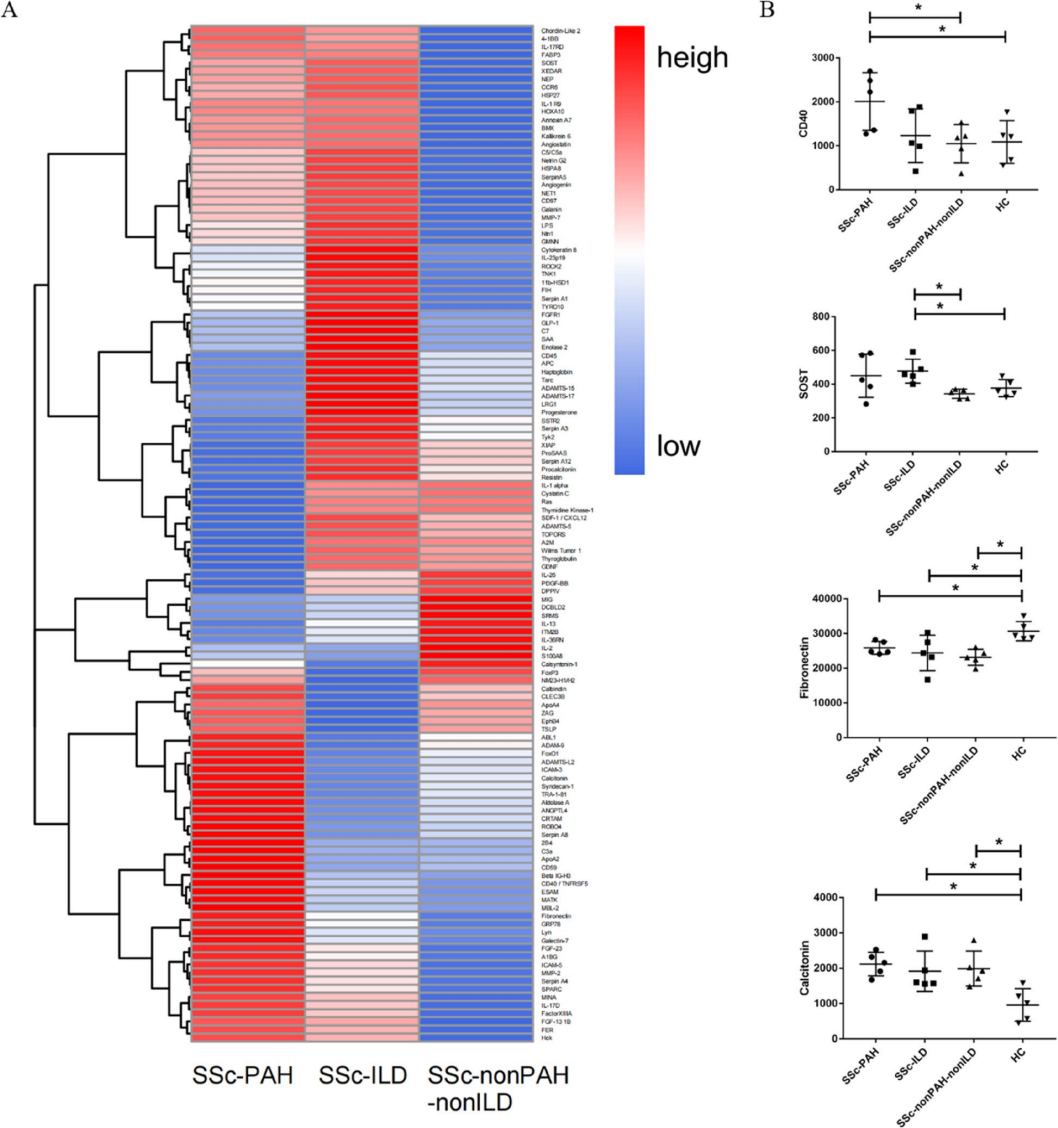
The expression of serum protein profiles in patients with SSc with different pulmonary complications using the high-throughput antibody microarray. **A** The heatmap of the log2 (fold change) of the 125 differentially expressed proteins in the microarray test. A total of 20 participants were equally divided into four groups, including the SSc with PAH (SSc-PAH), SSc with ILD (SSc-ILD), SSc without PAH and ILD (SSc-nonPAH-nonILD), and HC groups. A protein was considered differentially expressed when the *p*-value was < 0.05 and the fold change was > 1.2 or < 0.8. **B** Comparison of the four selected differentially expressed protein (CD40, SOST, fibronectin, and calcitonin) levels between each subgroup of patients with SSc and the HCs

Serum samples from 15 patients with SSc and five HCs were tested using an antibody microarray (RayBiotech, USA), which detected 1000 proteins (for detailed data, see Additional file 1). In this study, we screened out 125 DEPs from three SSc subgroups (59 DEPs from the SSc with PAH group, 26 DEPs from the SSc with ILD group, and 67 DEPs from the SSc without PAH and ILD group), compared with the HCs (Figs. 1 and 2A). Four specific proteins were selected as possible biomarkers of SSc or associated pulmonary complications for further validation. Because calcitonin and fibronectin were upregulated and downregulated, respectively, in all three SSc subgroups, they were chosen as possible biomarkers for SSc. Thus, CD40 was chosen as a possible biomarker for PAH in SSc because of its unique upregulation in the SSc with PAH group compared with the SSc without PAH and ILD group and HCs. SOST was chosen as a possible biomarker of ILD in SSc because of its unique upregulation in the SSc with the ILD group (Fig. 2B).

#### The GO analysis of differentially expressed proteins

The enormous amount of microarray data may reveal the intrinsic mechanism of SSc in addition to diagnostic biomarkers. We performed GO functional analysis for the top 10 biological activities in which the DEPs primarily participated for each SSc subgroup. GO analysis of the SSc with PAH group indicated that the processes of peptidyl-tyrosine phosphorylation, peptidyl-tyrosine modification, protein autophosphorylation, leukocyte migration, positive regulation of kinase activity, positive regulation of protein kinase activity, regulation of tissue remodeling, and cell chemotaxis were activated, and the processes of acute-phase response and negative regulation of endopeptidase activity were inhibited (Fig. 1A). For the SSc with ILD group, the processes of regulation of protein secretion, acute-phase response, positive regulation of secretion by cell, positive regulation of protein production, positive regulation of protein secretion, positive regulation of secretion, protein secretion, and establishment of protein localization to extracellular region were all activated (Fig. 1B). Finally, for the SSc without PAH and ILD group, the processes of acute inflammatory response, acute-phase response, negative regulation of hydrolase activity, complement-dependent cytotoxicity, and negative regulation of endopeptidase activity were inhibited, while the processes of positive regulation of protein production, humoral immune response, and regulation of lymphocyte proliferation were activated (Fig. 1C).

### Further validation and correlation analysis in SSc patients

#### Validation of differentially expressed proteins by ELISA

**Fig. 3:**
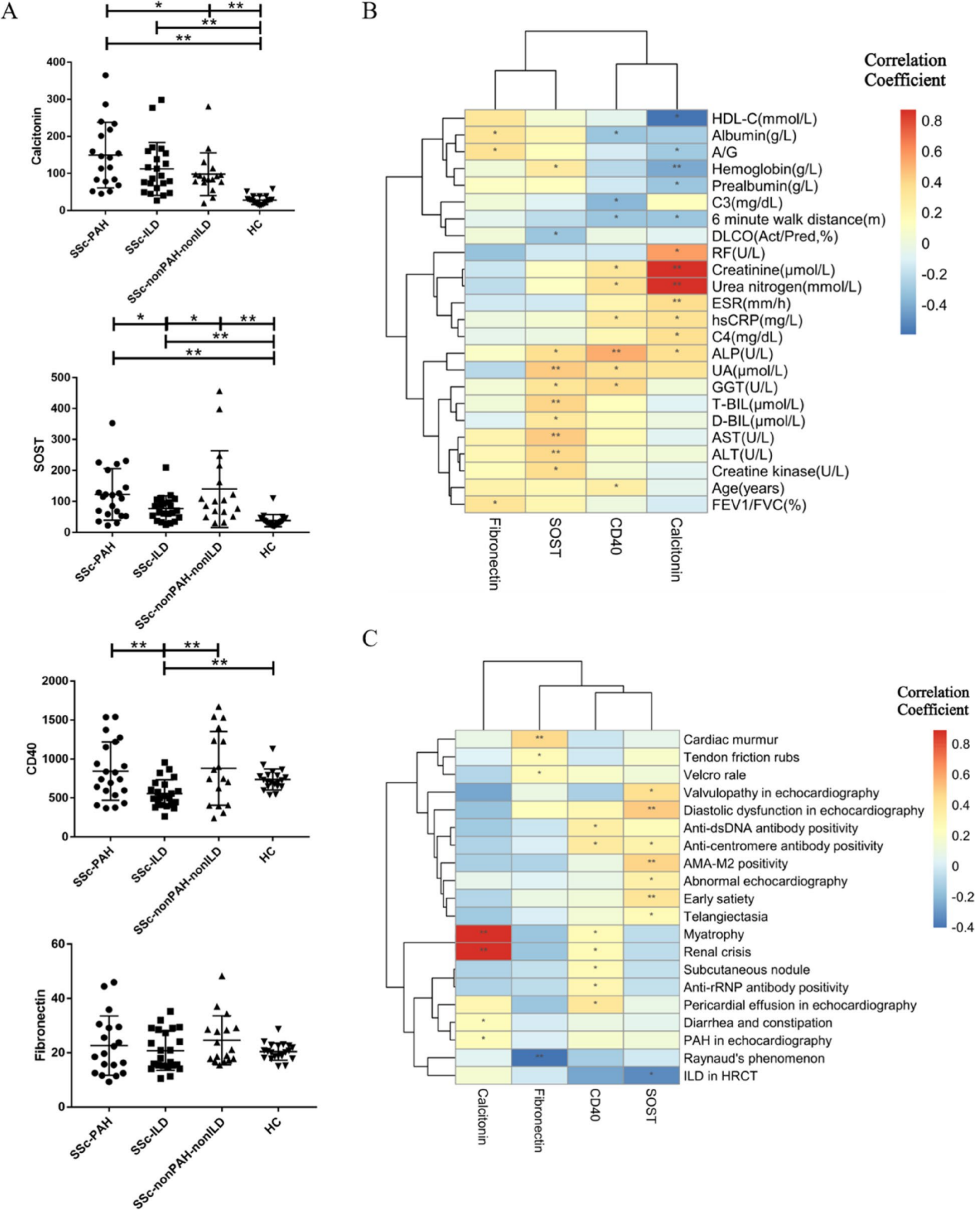
The validation and correlation analysis of the four selected proteins. **A** Comparison of serum levels of the four selected proteins between each SSc subgroup and the HCs using ELISA tests. **B**, **C** Correlation analysis of the clinical features/laboratory results/examination results and serum protein levels performed by the Pearson correlation analysis (**B**) and the point biserial correlation test (**C**). * indicated *p*-value < 0.05, ** indicated *p*-value < 0.01

ELISA was performed to validate the four selected DEPs in additional samples from treatment-naïve patients with SSc and HCs. This indicated that calcitonin and SOST serum levels were both significantly increased in all three SSc subgroups compared to the HCs (*n* = 20), while the calcitonin level was much higher in the SSc with PAH group (*n* = 19) and the SOST level was much lower in the SSc with ILD group (*n* = 22) than in the SSc without PAH and ILD group (*n* = 17). However, the CD40 level was not uniquely increased in the SSc with PAH group but was significantly decreased in the SSc with ILD group compared with the other SSc subgroups and HCs. Fibronectin levels did not differ between the groups (Fig. 3A).

#### Correlation study of protein levels and clinical characteristics

These four proteins were differentially expressed in patients with SSc in a protein microarray study. We also discovered that the correlation between SOST levels and the presence of ILD in SSc was controversial because SOST levels were negatively correlated with both the diffusing capacity of the lungs for carbon monoxide (DLCO%) and the presence of ILD on HRCT. Calcitonin levels may be positively correlated with the presence of PAH in SSc. In addition, calcitonin and CD40 levels might be positively related to renal damage in SSc because of their positive correlation with urea nitrogen levels, creatinine levels, and the presence of renal crisis. The SOST level was likely to be positively related to damage to liver cells, biliary system, gastrointestinal system, and myocardium, while calcitonin levels were likely to be positively related to gastrointestinal damage (Fig. 3B, C).

## Discussion

The causes of SSc-related deaths have changed over the past 50 years, possibly because of increasing knowledge about the pathogenic mechanism and proper treatment of SSc [3, 6, 7]. Currently, the main causes of death in patients with SSc, including SSc-related and SSc-unrelated deaths, are ILD (16.8%), PAH (14.7%), and cardiac disease (12.0%) [3]. Pulmonary complications are still the leading cause of death in SSc, whereas cardiac disease is an emerging cause of death, with a mortality ratio only second to pulmonary complications [3, 8]. Renal crisis was once the leading cause of death in patients with SSc, while it now accounts for only 2.9% of deaths [3, 6]. However, SSc renal crisis remains a critical internal complication that requires large doses of an angiotensin-converting enzyme inhibitor [9–11]. Gastrointestinal dysmotility is the most frequent complication of SSc and manifests as gastroesophageal reflux disease, dysphagia, early satiety, vomiting, diarrhea, and constipation [12–14]. Severe gastrointestinal disease in SSc correlates with high mortality, accounting for 3.9% of SSc deaths [12, 14]. Because of the poor prognosis, we aimed to identify specific biomarkers for the early detection of these major organ complications, especially pulmonary complications, in SSc.

Previous studies have investigated serum biomarkers for SSc pulmonary complications owing to the convenience of measuring them. For SSc-related ILD, it has been reported that high levels of anti-U11/U12 antibodies, human epididymis protein 4, secreted frizzled receptor protein 4, transcription factor scleraxis, endothelin-1, cold-inducible RNA-binding protein, Krebs von den Lungen-6, surfactant protein D, CA15-3, and intercellular adhesion molecule 1 were potential serum biomarkers for early detection and severity assessment of ILD [15–22]. Serum anti-U11/U12 antibodies are strongly associated with moderate-to-severe gastrointestinal dysmotility [22]. For SSc-related PAH, it has been reported that serum levels of anti-centromere antibody (especially the anti-p4.2 antibody subset), anti-vinculin antibody, IL-32, midkine, follistatin-like 3, osteopontin, chemerin, and specific long noncoding RNAs (e.g., ANCR and SPRY4-IT1) are positively correlated with the presence of PAH, whereas serum anti-topoisomerase antibody levels are negatively correlated with the presence of PAH [23–30]. In this microarray study, we identified 125 serum protein biomarkers in different pulmonary complication subgroups of patients with SSc. The selected four proteins (calcitonin, fibronectin, CD40, and SOST) were further verified by ELISA tests, among which the serum calcitonin level was confirmed to be upregulated in both SSc and SSc-PAH patients, and the serum SOST level was confirmed to be equally upregulated in each pulmonary complication subgroups of patients with SSc. Further analysis of the clinical data revealed a possible correlation between serum SOST levels and the presence of ILD. Moreover, serum CD40 and calcitonin levels appeared to be positively related to the presence of renal involvement, serum calcitonin, and SOST levels to the presence of gastrointestinal involvement, and serum SOST levels to the presence of cardiac involvement. The mechanisms underlying the differential expression of these proteins in SSc require further investigation.

The pathogenesis of SSc is characterized by endothelial injury, vasculopathy, tissue fibrosis, inflammation, and autoimmunity [31, 32]. Endothelial injury is proposed to be the initial event in SSc pathogenesis, whereas tissue fibrosis may be the culminating event [33]. Immune cells, including dendritic, Th2, and Treg cells, appear to be involved in both the early inflammatory response and the late fibrotic process [33]. These crucial events are regulated by the complex orchestration of genetic backgrounds, epigenetic modifications, environmental cues, molecular triggers, and intracellular signaling pathways [1, 32]. Bone metabolism appears to play a role in the pathogenesis of SSc; therefore, numerous skeletal disorders (including calcinosis, osteoporosis, and osteolysis) can occur in the early stages of SSc [34–36]. The pathogenesis of bone damage in SSc remains uncertain; however, microvascular dysfunction and hypoxic shock have been proposed as possible driving factors [34]. Both calcitonin and SOST are primarily considered bone metabolism regulators and were positively correlated with the presence of SSc and SSc-related pulmonary complications in this study. Calcitonin also participates in the regulation of extracellular matrix deposition, fibroblast activation, immune responses, inflammation, and endothelial dysfunction [37–41]. Additionally, SOST has been confirmed to be a Wnt signaling antagonist that is very important in SSc pathogenesis [1, 42]. SOST has also been reported to modulate immune cell development and differentiation of B, Th17, and Treg cells [43]. Therefore, serum calcitonin and SOST were proposed as biomarkers for patients with SSc and SSc-related pulmonary complications in this study. However, further verification is required because of the existence of controversial studies.

All patients with SSc recruited in this study were treatment-naïve; therefore, their serum protein profiles were undisturbed. PAH was diagnosed using a right heart catheter instead of echocardiography. Therefore, the results of this study are considered credible. However, this study has some limitations, such as the small sample size and lack of mechanistic studies. CD40 and fibronectin have been proposed to participate in the pathogenesis of SSc in previous studies, and their levels have been proposed to be elevated in patients with SSc [44–49]. However, there was no significant difference between the patients with SSc and HCs in this study. There are contradictory results regarding serum SOST levels in a few small-sample studies that showed no difference between patients with SSc and HCs [50, 51]. However, it has been suggested that serum SOST levels positively correlate with the severity of skin fibrosis [51]. Therefore, the results of this study need to be validated with more evidence from intensive studies.

## Conclusions

In conclusion, serum protein biomarkers may be useful for the early detection of vital organ involvement in SSc, which may lead to a poor prognosis. This study indicates that serum calcitonin and SOST levels are promising biomarkers for SSc-related PAH and ILD, respectively. Further research is needed to verify these results and understand the underlying mechanisms.

### Supplementary Information

**Additional file 1.** Serum samples from 15 patients with SSc and five HCs.

## Data Availability

The datasets used during the current study are available from the corresponding author on reasonable request.

## Abbreviations

SSc: Systemic sclerosis
PAH: Pulmonary arterial hypertension
ILD: Interstitial lung disease
SOST: Sclerostin
HC: Healthy control
GO: Gene Ontology
mPAP: Mean pulmonary arterial pressure
PAWP: Pulmonary arterial wedge pressure
HRCT: High-resolution computerized tomography
TRV: Tricuspid regurgitation velocity
DEP: Differentially expressed protein
DLCO: Diffusing capacity of the lungs for carbon monoxide
